# Vitamin D–Binding Protein Modifies the Vitamin D–Bone Mineral Density Relationship

**DOI:** 10.1002/jbmr.387

**Published:** 2011-03-17

**Authors:** Camille E Powe, Catherine Ricciardi, Anders H Berg, Delger Erdenesanaa, Gina Collerone, Elizabeth Ankers, Julia Wenger, S Ananth Karumanchi, Ravi Thadhani, Ishir Bhan

**Affiliations:** 1 Division of Nephrology, Department of Medicine, Massachusetts General Hospital and Harvard Medical SchoolBoston, MA, USA; 2 Clinical Research Center, Massachusetts Institute of TechnologyBoston, MA, USA; 3 Division of Clinical Pathology, Department of Clinical Chemistry, Beth Israel Deaconess Medical CenterBoston, MA, USA; 4 Howard Hughes Medical Institute and Division of Nephrology, Department of Medicine, Beth Israel Deaconess Medical Center and Harvard Medical SchoolBoston, MA, USA

**Keywords:** Vitamin D, Vitamin D–binding protein, Bone mineral density, Free hormone hypothesis, Vitamin D deficiency

## Abstract

Studies examining the relationship between total circulating 25-hydroxyvitamin D [25(OH)D] levels and bone mineral density (BMD) have yielded mixed results. Vitamin D–binding protein (DBP), the major carrier protein for 25(OH)D, may alter the biologic activity of circulating vitamin D. We hypothesized that free and bioavailable 25(OH)D, calculated from total 25(OH)D, DBP, and serum albumin levels, would be more strongly associated with BMD than levels of total 25(OH)D. We measured total 25(OH)D, DBP, and serum albumin levels in 49 healthy young adults enrolled in the Metabolic Abnormalities in College-Aged Students (MACS) study. Lumbar spine BMD was measured in all subjects using dual-energy X-ray absorptiometry. Clinical, diet, and laboratory information also was gathered at this time. We determined free and bioavailable (free + albumin-bound) 25(OH)D using previously validated formulas and examined their associations with BMD. BMD was not associated with total 25(OH)D levels (*r* = 0.172, *p* = .236). In contrast, free and bioavailable 25(OH)D levels were positively correlated with BMD (*r* = 0.413, *p* = .003 for free, *r* = 0.441, *p* = .002 for bioavailable). Bioavailable 25(OH)D levels remained independently associated with BMD in multivariate regression models adjusting for age, sex, body mass index, and race (*p* = .03). It is concluded that free and bioavailable 25(OH)D are more strongly correlated with BMD than total 25(OH)D. These findings have important implications for vitamin D supplementation in vitamin D–deficient states. Future studies should continue to explore the relationship between free and bioavailable 25(OH)D and health outcomes. © 2011 American Society for Bone and Mineral Research.

## Introduction

Vitamin D deficiency is associated with decreased calcium absorption and elevated levels of parathyroid hormone (PTH),^1^ which may lead to excessive bone resorption.^2^ In some observational studies, higher levels of 25-hydroxyvitamin D [25(OH)D] have been linked to increased bone mineral density (BMD) and decreased risk of fracture.^3^–^7^ Additionally, several randomized, controlled trials suggest that vitamin D supplementation reduces the risk of fracture and increases BMD.^8^–^14^ However, not all observational studies have confirmed the relationship between 25-hydroxyvitamin D [25(OH)D] and BMD, especially in younger populations or racial minorities.^15^–^19^ Moreover, in several randomized trials, the effect of vitamin D supplementation on BMD or fracture risk has been modest,^8^ absent,^20^–^22^ or reversed.^23^

The free hormone hypothesis postulates that only hormones liberated from binding proteins enter cells and produce biologic action.^24^ 25(OH)D and 1,25-dihydroxyvitamin D_3_ [1,25(OH)_2_D_3_] circulate bound to vitamin D–binding protein (85% to 90%) and albumin (10% to 15%), with less than 1% of circulating hormone in its free form.^25^ In mice, vitamin D–binding protein (DBP) prolongs the serum half-life of 25(OH)D and protects against vitamin D deficiency by serving as a vitamin D reservoir.^26^ However, DBP also limits the biologic activity of injected 1,25(OH)_2_D_3_ in mice^26^ and inhibits the action of vitamin D on monocytes and keratinocytes in vitro.^27^, ^28^ The significance of circulating DBP levels with regard to vitamin D's biologic action in humans is unclear.

The free fraction of 25(OH)D and the binding-affinity constants for 25(OH)D's interaction with DBP and albumin have been measured previously.^29^ Formulas for the calculation of free 25(OH)D levels based on serum concentrations of total 25(OH)D, DBP, and albumin have been developed based on these data. Measured and calculated values of free 25(OH)D are highly correlated.^29^ To determine whether DBP modifies the relationship between 25(OH)D levels and BMD, we measured serum levels of total 25(OH)D, DBP, and albumin in a group of healthy young adults and calculated levels of free and bioavailable (free + albumin bound) 25(OH)D using adaptations of previously validated formulas. We then determined whether free or bioavailable 25(OH)D levels are more strongly associated with lumbar spine BMD than total 25(OH)D levels in this population.

## Materials and Methods

### Subject recruitment

We conducted a cross-sectional study in a subset of healthy young adults enrolled in the Metabolic Abnormalities in College Students (MACS) study, a study designed to evaluate the prevalence of metabolic abnormalities in university students.^30^ Subjects were healthy 18- to 31-year-old male and female students from private universities in the Boston area. A total of 170 subjects were recruited through flyers posted throughout the Massachusetts Institute of Technology (MIT) campus and through targeted e-mails to random members of the student population. All subjects provided written informed consent. The study was approved by the MIT Committee on the Use of Humans as Experimental Subjects. Forty-nine subjects had sufficient sample for inclusion in this analysis, and their characteristics are presented in Table 1.

**Table 1:** Characteristics of the Study Population (*n* = 49)

	Mean ± SD, *n* (%)
Age (years)	23.5 ± 3.4
Body mass index (kg/m^2^)	22.43 ± 2.96
Sex	
Male	27 (55.1%)
Female	22 (44.9%)
Race	
White	31 (63.3%)
Nonwhite	18 (36.7%)
Exercise amount	
>120 minutes per week	21 (42.9%)
≤120 minutes per week	26 (53.1%)
Unknown	2 (4.1%)
Vitamin D–binding protein (µmol/L)	4.19 ± 2.49
Albumin (g/L)	42.47 ± 3.94
Serum calcium (mmol/L)	2.30 ± 0.19
Parathyroid hormone (ng/L)	29.86 ± 8.25
Dietary calcium intake (mg/d)	925.85 ± 421.76
Lumbar spine BMD (g/cm^2^)	1.05 ± 0.14

*Note:* Data are presented as *n* (%) for categorical variables and mean ± SD for continuous variables.

### Study visit

Subjects were instructed to fast for 12 hours prior to admission to the MIT Clinical Research Center (CRC) as outpatients and underwent a baseline evaluation including a blood sample collection and various physiologic measurements. Structured interviews were conducted by study nurses to collect standard clinical information, minutes of exercise per week (in 30-minute increments), and medication/supplement use. Height was measured using a standing stadiometer (Holton, Ltd., Crymych, Dyfed, UK). Weight was measured using a calibrated scale (SECA, Hanover, MD, USA). Body mass index (BMI) was calculated as weight (kg)/[height (m)]^2^.

### Dietary information

Subjects completed a written food record 1 week prior to the day of study, recording 4 full days of food intake, including one weekend day. During the study visit to the MIT CRC, a registered dietitian reviewed the food record with the subject to clarify the quantities and sources of food consumed. Dietary intake data then were analyzed using Nutrition Data System for Research software version 2006/2007 (Nutrition Coordinating Center, University of Minnesota, Minneapolis, MN, USA).

### Bone density measurement

Subjects underwent total-body dual-energy X-ray absorptiometry (DXA; Hologic QDR-4500A, Hologic, Waltham, MA, USA) to determine total and regional BMD.^31^ Hologic phantoms were used to calibrate the instrument. We used lumbar spine BMD in this study as the measure of BMD. Lumbar spine BMD is a preferred site for the diagnosis of osteoporosis and the prediction of fracture. No hip BMD measurements were available.^32^, ^33^

### Biochemical analysis

Baseline blood samples were frozen at −80°C and stored for later analysis. 25(OH)D, serum calcium, albumin, and levels of parathyroid hormone (PTH) were measured in the Massachusetts General Hospital (MGH) clinical laboratories. 25(OH)D_2_ and 25(OH)D_3_ levels were measured by liquid chromatography–tandem mass spectrometry (LC-MS), with interassay coefficients of variation (CVs) of 9.1% for 25(OH)D_2_ and 8.6% for 25(OH)D_3_. Total 25(OH)D level was calculated as the sum of 25(OH)D_2_ level and 25(OH)D_3_ level. Intact PTH was measured by electrochemiluminescense immunoassay on the Cobas E160 automated analyzer (Roche Diagnostics, Indianapolis, IN, USA). The interassay CV for intact PTH measurement was 4.2%. Calcium and albumin levels were measured by dye-based photometric assays on an automated analyzer.

DBP was measured in duplicate by commercial enzyme-linked immunosorbent assay (ELISA; Cat. No. DVDBP0, R&D Systems, Minneapolis, MN, USA) according to the manufacturer's instructions. The assay was conducted after diluting serum samples 1:2000 in Calibrator Diluent RD6-11 (Part No. 895489, R&D Systems). The interassay CV was 8.5% at a concentration of 40 µg/mL. The assay recovered between 93% and 110% of a 100- to 200-µg/mL dose of exogenous DBP added to human serum samples containing between 25 and 200 µg/mL of endogenous DBP. The manufacturer reports no significant cross-reactivity with human albumin, vitamin D_3_, or α-fetoprotein.

In a subset of patients in whom adequate serum was available (*n* = 45), total 1,25(OH)_2_D_3_ was measured by LC-MS/MS in the Mayo Clinic Medical Laboratories (Rochester, MN, USA).

### Calculation of unbound 25(OH)D

Free levels of 25(OH)D were calculated using two methods. Both methods used the binding-affinity constants between albumin and DBP and 25(OH)D measured in a previous study that used centrifugal ultrafiltration to determine the free fraction of 25(OH)D.^29^

#### Method 1

Free levels of 25(OH)D were calculated using the following equation^29^:


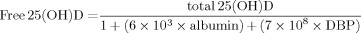


The reported correlation coefficient between calculated free 25(OH)D using this equation and measured free 25(OH)D by centrifugal ultrafiltration is 0.925.^29^ Free 1,25(OH)_2_D_3_ levels also were calculated using this method.^25^

#### Method 2

Free, bioavailable, and DBP-bound 25(OH)D were calculated using equations adapted from those described by Vermeulen and colleagues.^34^ These methods define bioavailable hormone as the fraction that is both free and albumin-bound, that is, the fraction not bound to circulating binding proteins such as DBP. This method has been validated for calculation of free and bioavailable testosterone based on measured amounts of serum total testosterone, sex hormone–binding globulin (SHBG), and albumin and application of the known binding-affinity constants of testosterone for albumin and SHBG. For this study, the Vermeulen equations were adapted by replacing the variables for testosterone, SHBG, and albumin and their respective binding constants with those of 25(OH)D, DBP, and albumin. The resulting formulas and an example calculation of free and bioavailable 25(OH)D are shown in the Supplementary Material.

Both calculation methods used the same affinity-binding constants. Applied to the same measurements of total 25(OH)D, DBP, and albumin, they produce calculated free 25(OH)D values that are highly correlated (Spearman *r* = 1), but the modified Vermeulen equations produce values that are an average of 1.4% higher (data not shown). Because the modified Vermeulen equations also provide for separate calculation of free, bioavailable, and DBP-bound 25(OH)D, we used the modified Vermeulen methods (*Method 2*) for subsequent analyses of 25(OH)D levels.

### Statistical analysis

Subject characteristics are reported as mean ± SD unless otherwise noted. Nonnormal variables, including 25(OH)D levels, DBP levels, BMD, and dietary calcium intake levels, showed skewed distributions and were natural-log-transformed in order to meet the assumptions of parametric statistical techniques. Exercise amount was dichotomized at 120 minutes per week. Pearson's correlation coefficients were calculated to assess the relationships between 25(OH)D levels, BMD, and other continuous variables. Independent-samples *t* tests were used to compare 25(OH)D levels, DBP levels, and BMD among subgroups defined by race, sex, exercise amount, and oral contraceptive use. Linear regression analysis was used to test for the presence of an independent relationship between 25(OH)D levels, DBP, and BMD after adjustment for factors previously reported to be associated with bone density, including age, sex, BMI, and race.^5^, ^8^, ^12^, ^35^, ^36^ All analyses were conducted using STATA Statistical Software Version 11 (College Station, TX, USA). Two-sided *p* values < .05 were considered statistically significant.

## Results

Subject characteristics are shown in Table 1. There was wide variation in levels of DBP, with concentrations ranging from 0.66 to 11.2 µmol/L. Accordingly, calculated free and bioavailable 25(OH)D levels ranged widely (Table 2). Total 25(OH)D levels were positively correlated with DBP levels (*r* = 0.335, *p* = .019).

**Table 2:** Serum Levels of Vitamin D

	Mean ± SD
Total 25(OH)D (nmol/L)	64.23 ± 27.70
DBP-bound 25(OH)D (nmol/L)	54.66 ± 26.32
Albumin-bound 25(OH)D (nmol/L)	9.55 ± 6.72
Free 25(OH)D (pmol/L)	25.37 ± 18.52
Bioavailable 25(OH)D (nmol/L)	9.58 ± 6.74

*Note:* Total 25(OH)D levels were measured along with albumin and DBP. DBP-bound, albumin-bound, free, and bioavailable 25(OH)D (free and albumin-bound) were calculated using equations adapted from Vermeulen.^34^

**Fig. 1:**
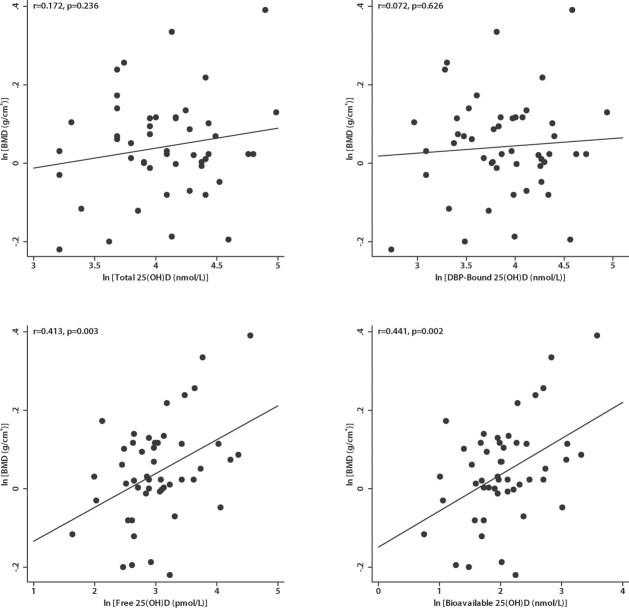
Relationship between total and free 25(OH)D and lumbar spine BMD. DBP-bound, free, and bioavailable 25(OH)D levels were calculated from measured total 25(OH)D and DBP levels using equations adapted from Vermeulen.^34^ Total 25(OH)D and DBP-bound 25(OH)D were not associated with lumbar spine BMD. Free 25(OH)D and bioavailable 25(OH) D were positively correlated with lumbar spine BMD.

Total 25(OH)D levels were not correlated with BMD (*r* = 0.172 *p* = .236; Fig. 1). Similarly, levels of DBP-bound 25(OH)D were not correlated with BMD (*r* = 0.072, *p* = .626). In contrast, free and bioavailable 25(OH)D levels were both strongly correlated with BMD (*r* = 0.413, *p* = .003 for free and *r* = 0.441, *p* = .002 for bioavailable; Fig. 1). Bioavailable and free 25(OH)D levels were highly correlated with each other (*r* = 0.985, *p* < .001), but bioavailable 25(OH)D made up a larger portion of the total 25(OH)D, with approximately 350-fold higher concentrations of bioavailable 25(OH)D compared with free 25(OH)D (Table 2). Total and calculated free levels of 1,25(OH)_2_D_3_ were not correlated with BMD (*p* > .05). Total levels of 1,25(OH)_2_D_3_ were not associated with free or bioavailable 25(OH)D levels (*p* > .05), nor was sex-adjusted alkaline phosphatase associated with total, free, or bioavailable 25(OH)D (*p* > .05) Neither total nor bioavailable 25(OH)_2_D_3_ levels were correlated with serum calcium or PTH levels (*p* > .05). Of note, PTH levels fell between 15 and 51 ng/L (all within the normal range) and were not associated with BMD (*r* = −0.024, *p* = .869).

Both total 25(OH)D and DBP levels were inversely associated with BMI (*r* = −0.300, *p* = .036 for total and *r* = −0.542, *p* < .001 for DBP). Bioavailable 25(OH)D was positively correlated with BMI (*r* = 0.302, *p* = .035). However, in this population, BMI was not correlated with BMD (*r* = 0.160, *p* = .271). Dietary calcium intake was correlated with total 25(OH)D (*r* = 0.339, *p* = .021) but was not correlated with DBP, bioavailable 25(OH)D, or BMD (*p* > .05). Levels of total and bioavailable 25(OH)D, DBP, and BMD among selected subgroups are shown in Table 3. Females had greater average total 25(OH)D levels than males, but average DBP levels, bioavailable 25(OH)D levels, and BMD did not differ between males and females. Females reporting use of oral contraceptive pills (OCPs) had higher average total 25(OH)D levels than females who did not report OCP use, but average DBP and bioavailable 25(OH)D levels were not significantly different based on OCP use. Subjects with BMI greater than or equal to 25 kg/m^2^ (overweight subjects) had lower DBP levels than subjects with BMI less than 25 kg/m^2^ (normal-weight subjects). Subjects who reported exercising 120 minutes a week or more had higher average total 25(OH)D levels than subjects who did not, but no significant difference was found in average DBP levels, bioavailable 25(OH)_2_D_3_ levels, and BMD. Average DBP levels in nonwhite subjects were lower than in white subjects (Table 3).

**Table 3:** BMD, DBP Levels, and 25(OH)D Levels in Select Subgroups

	*n*	Total 25(OH)D (nmol/L)	DBP (µmol/L)	Bioavailable 25(OH)D (nmol/L)	L-spine BMD (g/cm^2^)
Sex					
Male	27	52.79 ± 19.31	3.90 ± 2.09	8.36 ± 5.36	1.04 ± 0.14
Female	22	78.28 ± 30.28	4.53 ± 2.92	11.08 ± 8.01	1.07 ± 0.13
*p* Value		<.001	.493	.113	.371
OCP use (females only)					
Yes	7	107.33 ± 27.87	6.27 ± 3.76	12.83 ± 11.64	1.06 ± 0.21
No	15	64.73 ± 20.59	3.71 ± 2.12	10.26 ± 5.98	1.07 ± 0.08
*p* Value		<.001	.152	.841	.646
Body mass index					
<25 kg/m^2^	39	66.0 ± 26.9	4.63 ± 2.49	8.59 ± 5.28	1.04 ± 0.12
≥25 kg/m^2^	10	57.2 ± 25.0	2.42 ± 1.61	13.43 ± 10.19	1.09 ± 0.19
*p* Value		.284	.003	.073	.438
Exercise					
≥120 minutes/week	21	73.33 ± 26.11	4.14 ± 2.56	11.76 ± 8.35	1.09 ± 0.13
*<*120 minutes/week	26	58.66 ± 27.97	4.20 ± 2.58	8.19 ± 4.85	1.03 ± 0.13
*p* Value		.038	.863	.089	.165
Race					
White	31	68.84 ± 28.65	4.94 ± 2.43	7.84 ± 3.92	1.03 ± 0.10
Nonwhite	18	56.30 ± 24.76	2.87 ± 2.04	12.56 ± 9.29	1.08 ± 0.18
*p* Value		.138	<.001	.065	.346

*Note:* Values are reported as mean ± SD. DBP = vitamin D–binding protein; BMD = bone mineral density; OCP = oral contraceptive pill. Groups were compared using *t* tests after natural-log transformation of total 25(OH)D, DBP, bioavailable 25(OH)D levels, and BMD.

In multivariate models adjusting for age, sex, BMI, and race, bioavailable 25(OH)D remained independently associated with BMD (*p* = .03; Table 4). Bioavailable 25(OH)D was the only significant predictor of BMD in multivariate models. Since the level of calculated bioavailable 25(OH)D depends on the concentrations of total 25(OH)D, albumin, and DBP, we separately assessed whether albumin or DBP was associated with BMD. DBP level was inversely correlated with BMD (*r* = −0.296, *p* = .039), whereas serum albumin showed no association with BMD (*r* = 0.156, *p* = .285). In a multivariate linear regression model, total 25(OH)D became a significant predictor of BMD only after adjustment for DBP level (*B* = 0.089, *p* = .040). Albumin was not associated with BMD in a multivariate model including DBP and total 25(OH)D (*p* = .150).

**Table 4:** Bioavailable 25(OH)D Predicts BMD

Model	B	*p* Value	Adjusted *R*^2^
Bioavailable 25(OH)D	0.092	0.002	0.177
Bioavailable 25(OH)D, age, sex, race, and BMI	0.072	0.029	0.180

*Note:* Bioavailable 25(OH)D and BMD were natural-log-transformed prior to analysis. The coefficient (B) represents the average unit increase in ln BMD for each unit increase in ln bioavailable 25(OH)D. *p* Value is the statistical significance of the relationship between bioavailable 25(OH)D and BMD after controlling for potential confounders. Thus the relationship between bioavailable 25(OH)D and BMD remains significant after adjusting for potential confounders.

## Discussion

In light of conflicting reports concerning the relationship between circulating levels of 25(OH)D and BMD, we measured serum levels of total 25(OH)D, DBP, and albumin in a group of young, healthy adults and assessed relationships between free 25(OH)D, bioavailable 25(OH)D, total 25(OH)D, and BMD. Consistent with the free hormone hypothesis, the results of our study suggest that circulating DBP is an inhibitor of the biologic action of vitamin D in humans. The similar associations between free and bioavailable vitamin levels and BMD imply that unlike binding to DBP, binding to albumin does not inhibit the action of 25(OH)D. These results are consistent with prior basic and clinical studies on DBP. The inverse relationship between DBP levels and BMD demonstrated here is in agreement with prior in vitro work finding inhibitory effects of DBP on 25(OH)D and 1,25(OH)_2_D_3_ action on monocytes and keratinocytes.^27^, ^28^ A modified form of DBP also may have direct activating effects on osteoclasts; this also could explain the inverse relationship between DBP and BMD.^37^ Recent reports have described humans with genetic polymorphisms in the *DBP* gene that were associated with corresponding changes in total 25(OH)D levels and risk of osteoporosis.^38^–^43^ This suggests that DBP may be an important regulator of vitamin D pharmacokinetics, metabolism, and action. Measured levels of 25(OH)D and DBP in our study were positively correlated, an observation that combined with the genetic studies leads us to speculate that total circulating levels of 25(OH)D may be determined in part by DBP levels. Of note, PTH levels were not associated with 25(OH)D levels or BMD, suggesting that the association between free or bioavailable 25(OH)D levels and BMD is not mediated via PTH. Although we did not find an independent association between alkaline phosphatase and any form of vitamin D, future studies should assess additional markers of bone turnover, particularly following interventions to alter free or total vitamin D levels.

The results of this study support the hypothesis that DBP behaves similarly to other serum hormone carrier proteins and have broad clinical applications. Like thyroid hormone–binding globulin and sex hormone–binding globulin, DBP may act as a serum carrier and reservoir, prolonging the circulating half-life of vitamin D while at the same time regulating its immediate bioavailability to target tissues.^24^ In contrast to the megalin-mediated endocytosis described in renal tubular cells, our results imply that 25(OH)D gains access to some target cells by diffusion across cell membranes, similar to these other steroid hormones.^24^ Thus hormonal activity and sufficiency may be reflected by the amounts of bioavailable vitamin, not by total serum levels. Currently, clinical testing for vitamin D deficiency is based on measurement of total serum concentrations of 25(OH)D.^2^ Yet our data suggest that concentrations of total serum vitamin D may not be the best measure of vitamin D sufficiency. For example, patients with high levels of DBP may appear to be 25(OH)D-sufficient but actually may be deficient in bioavailable vitamin. Conversely, in patients with low levels of DBP, total 25(OH)D will be low, but these patients actually may have sufficient bioavailable vitamin. The maintenance of bioavailable 25(OH)D levels in obese and nonwhite subjects, despite lower levels of total 25(OH)D, raise the possibility that variation in circulating DBP explains the apparent paradox of low 25(OH)D levels and higher BMD in black and overweight patients seen in several previous studies,^5^, ^15^, ^16^, ^35^, ^44^, ^45^ although this hypothesis requires further testing.

Our results contrast with results of some prior studies linking total 25(OH)D levels to BMD but are consistent with other studies that failed to find such a relationship.^4^, ^5^, ^15^–^18^ In these prior studies, DBP levels were not measured. Of note, total 25(OH)D and free/bioavailable 25(OH)D levels are associated, and it is possible that a larger sample size would have enabled us to detect a weak relationship between total 25(OH)D and BMD. Prior studies that found this relationship generally had sample sizes greater than 200, and when correlation coefficients between 25(OH)D and BMD were reported, they were less than 0.2.^4^, ^5^, ^16^, ^19^

We did not find a relationship between 1,25(OH)_2_D_3_ levels and BMD. While 1,25(OH)_2_D_3_ is thought to be the active form of vitamin D, many tissues express 1α-hydroxylase and may be able to convert circulating 25(OH)D to its active form locally.^46^ Circulating 25(OH)D levels generally are considered to better reflect overall vitamin D stores.^2^ Our results are in agreement with this, suggesting that total circulating total or free 1,25(OH)_2_D_3_ levels are not good measures of vitamin D activity. This is analogous to the accepted model for the measurement of thyroid hormone action, where free T_4_ levels are a better measure of thyroid hormone action than circulating free T_3_ levels, even though T_3_ is the active form of the hormone.^47^

This was a cross-sectional, observational study; consequently, we are unable to make conclusions about potential causal associations between vitamin D levels, DBP levels, and BMD. It is possible that the relationship between bioavailable 25(OH)D levels and BMD is due to some unknown confounder. However, we were unable to identify such a confounder in our analyses, and multivariate adjustment suggested that the relationship between free 25(OH)D and BMD was independent of such factors as sex, race, and BMI. The equations used to calculate free and bioavailable 25(OH)D levels have not been validated using the assays for DBP and 25(OH)D levels used in this study. However, the inverse associations between DBP and BMD did not depend on these calculations. Because of their low concentrations, there are significant technical challenges in direct measurement of free vitamin D levels. While some investigators have performed either centrifugal ultrafiltration or symmetric dialysis methods, these are prone to poor assay standardization and laboratory bias.^25^, ^29^, ^48^ The use of standardized immunoassays for vitamin D, DBP, and albumin combined with standard calculation methods would allow our approach to be adopted with more confidence by other clinical laboratories. Regardless, additional studies examining binding characteristics of 25(OH)D using these methods may allow further refinement of the formulas presented here.

We found a wide distribution of DBP levels among our subjects and observed that DBP was negatively associated with both high BMI and black race, both of which have been associated with low 25(OH)D levels. One potential explanation is that 25(OH)D may itself regulate the production of DBP. Lowering DBP levels would allow a higher fraction of DBP to be bioavailable in situations where total levels are low. Other possible explanations for the observed associations between race, BMI, and DBP levels include genetic factors and uptake of circulating DBP by adipose tissue. Future studies may shed light on the factors that regulate DBP.

This study provides evidence that DBP modifies the relationship between 25(OH)D and BMD in humans. Our data suggest that bioavailable 25(OH)D levels are a better of measure of vitamin D activity than total 25(OH)D levels, at least with respect to bone metabolism. It is therefore possible that by using total 25(OH)D levels as a measure of vitamin D sufficiency, individuals may be misclassified as sufficient or insufficient in vitamin D. This may explain conflicting results of prior studies of the relationship between serum 25(OH)D concentrations and BMD. Determining which individuals have a true deficit in vitamin D may allow future vitamin D supplementation interventions to be targeted to individuals most likely to benefit. Additionally, use of bioavailable 25(OH)D levels may further elucidate the nature of the relationship between vitamin D and a wide range of outcomes including fracture,^7^ infection,^49^ cancer,^50^ and cardiovascular disease.^51^
