# Supplementary material for: Vitamin D–Binding Protein Modifies the Vitamin D–Bone Mineral Density Relationship
Source: J Bone Miner Res. 2011 Mar 17;26(7):1609–16. doi: 10.1002/jbmr.387 (PMC3351032; doi:10.1002/jbmr.387)
Supplement: Supplementary file 1 [file jbmr0026-1609-SD1.pdf]

Supplementary Appendix 1. Derivation of Calculated Free and Bioavailable 25-hydroxyvitamin D from Vermuelen et al.

DEFINITIONS

D = 25-hydroxyvitamin D (calcidiol), sum of both D2 and D3

Alb = albumin

DBP = Vitamin D binding protein, also known as Group-specific component or Gc

$[D_{Alb}]$ = concentration of albumin-bound vitamin D

$[D_{DBP}]$ = concentration of DBP-bound vitamin D

[D] = concentration of free (unbound) D

[Total] = concentration of Total 25OH-D = $[D_{DBP}] + [D_{Alb}] + [D]$

[Bio] = concentration of Bioavailable D (Bioavailable = sum of free and albumin-bound vitamin) = $[D] + [D_{Alb}]$

K_{alb} = affinity constant between vitamin D and albumin = $6 \times 10^5\ M^{-1}$

K_{DBP} = affinity constant between vitamin D and DBP = $0.7 \times 10^9\ M^{-1}$

EQUATIONS

Total 25(OH)-Vitamin D

[Total] = concentration of 25(OH)-Vitamin D in g/mol ÷ 400.5 g/mole

Given that $[Total] = [D] + [D_{Alb}] + [D_{DBP}]$

thus $[D_{DBP}] = [Total] - [D_{Alb}] – [D]$ (Eq. 1)

Albumin

[Alb] = serum albumin concentration in g/L ÷ 66,430 g/mole

$[D] + [Alb] \leftrightarrow [D_{Alb}]$

Albumin association constant $K_{alb} = [D_{Alb}] \div ([D] \cdot [Alb])$

Thus $[D_{Alb}] = K_{alb} \cdot [Alb] \cdot [D]$ (Eq. 2)

(**NB**: [Alb] in this example denotes the concentration of free non-vitamin bound albumin. However, given the low affinity between albumin and Vit. D, the concentrations of total albumin and unbound albumin are effectively equivalent ([Total Albumin] $\approx$ [Alb]). As a result, [Alb] may be estimated accurately by measurements of total serum albumin.)

DBP

[Total DBP] = concentration of serum DBP in g/L $\div$ 58,000 g/mole

[DBP] = free unbound DBP *and* $[D_{DBP}]$ = vitamin-bound DBP

Given that $[D] + [DBP] \leftrightarrow [D_{DBP}]$

And DBP association constant $K_{DBP} = [D_{DBP}] \div ([DBP] \cdot [D])$

Thus $[D] = [D_{DBP}] \div K_{DBP} \div [DBP]$ (Eq. 3)

Since [Total DBP] = sum of bound and unbound DBP = $[DBP] + [D_{DBP}]$

Therefore $[DBP] = [Total\ DBP] - [D_{DBP}]$ (Eq. 4)

Solving for Free 25(OH)-Vitamin D

From Eqs. 3 and 4 we see that:

$$[D] = [D_{DBP}] \div K_{DBP} \div ([Total\ DBP] - [D_{DBP}]) \quad \text{(Eq. 5)}$$

If we substitute Eq. 1 into Eq. 2, we find that:

$$[D_{DBP}] = [Total] - (K_{alb} \cdot [Alb] + 1) \cdot [D] \quad \text{(Eq. 6)}$$

Substituting Eq. 6 into Eq. 5 produces the following:

$$[D] = \{[Total] - (K_{alb} \cdot [Alb] + 1) \cdot [D]\} \div K_{DBP} \div ([Total\ DBP] - \{[Total] - (K_{alb} \cdot [Alb] + 1) \cdot [D]\})$$

The equation is now limited to known constants (K_{DBP} and K_{alb}), measured values ([Total DBP], [Alb], and [Total]) and the dependent variable for free vitamin D [D]. After propagating products and several rearrangements we can further simplify this to fit the form of a second-degree polynomial:

$$ax^2 + bx + c = 0$$

Where x = [D] = the concentration of free 25(OH)-Vitamin D

$a = K_{DBP} \cdot K_{alb} \cdot [Alb] + K_{DBP}$

b = $K_{DBP} \cdot$ [Total DBP] – $K_{DBP} \cdot$ [Total] + $K_{alb} \cdot$ [Alb] +1

c = -[Total]

This polynomial may be solved for [D] using the quadratic equation:

$$[D] = [-b+\sqrt{b^2 - 4ac}] \div 2a$$

After solving for free 25(OH)-vitamin D, we may then use Eq. 2 to calculate the concentration of bioavailable (non-DBP bound vitamin):

$$[Bio] = [D] + [D_{Alb}] = (K_{alb} \cdot [Alb] + 1) \cdot [D] \qquad \text{(Eq. 7)}$$

EXAMPLE CALCULATION

Total 25(OH)-vitamin D = [Total] = 40 ng/mL = 1.0×10^{-7} mol/L

Total serum DBP = [Total DBP] = 250 ug/mL = 4.3×10^{-6} mol/L

Total serum albumin = [Alb] = 4.3 g/dL = 6.4×10^{-4} mol/L

$K_{alb} = 6 \times 10^{5}\ M^{-1}$

$K_{DBP} = 7.0 \times 10^{8}\ M^{-1}$

a = 2.7×10^{11}

b = 3325

c = $- 1 \times 10^{-7}$

Calculated concentration of free 25(OH)D = 3.01×10^{-11} mol/L = 12.1 pg/mL

Calculated concentration of bioavailable 25(OH)D = 1.09×10^{-8} mol/L = 4.6 ng/mL
